# Acute Kidney Injury due to Anticoagulant-Related Nephropathy : A Suggestion for Therapy

**DOI:** 10.1155/2020/8952670

**Published:** 2020-06-08

**Authors:** Letizia Zeni, Chiara Manenti, Simona Fisogni, Vincenzo Terlizzi, Federica Verzeletti, Mario Gaggiotti, Giovanni Cancarini

**Affiliations:** ^1^University of Campania Luigi Vanvitelli, Naples, Italy; ^2^Fondazione Policlinico Universitario A. Gemelli IRCCS, O. U. of Nephrology, Rome, Italy; ^3^O. U. of Nephrology, ASST Spedali Civili Brescia, Brescia, Italy; ^4^Nephrology, Department of Medical and Surgical Specialties, Radiological Science and Public Health, University of Brescia, Brescia, Italy

## Abstract

The relationship between kidneys and anticoagulation is complex, especially after introduction of the direct oral anticoagulants (DOAC). It is recently growing evidence of an anticoagulant-related nephropathy (ARN), a form of acute kidney injury caused by excessive anticoagulation. The pathogenesis of kidney damage in this setting is multifactorial, and nowadays, there is no established treatment. We describe a case of ARN, admitted to our Nephrology Unit with a strong suspicion of ANCA-associated vasculitis due to gross haematuria and haemoptysis; the patient was being given dabigatran. Renal biopsy excluded ANCA-associated vasculitis and diagnosed a red blood cell cast nephropathy superimposed to an underlying IgA nephropathy. Several mechanisms are possibly responsible for kidney injury in ARN: tubular obstruction, cytotoxicity of heme-containing molecules and free iron, and activation of proinflammatory/proﬁbrotic cytokines. Therefore, the patient was given a multilevel strategy of treatment. A combination of reversal of coagulopathy (i.e., withdrawal of dabigatran and infusion of its specific antidote) along with administration of fluids, sodium bicarbonate, steroids, and mannitol resulted in conservative management of AKI and fast recovery of renal function. This observation could suggest a prospective study aiming to find the best therapy of ARN.

## 1. Introduction

Despite initial scepticism, anticoagulant-related nephropathy is now considered in the lexicon of nephrologists. If suggestions for diagnostic pathway are available [1], a standardised strategy of treatment is still lacking. This report is intended to describe a case of biopsy-proven dabigatran-related nephropathy superimposed to undiagnosed IgA nephropathy and discuss a possible therapy.

## 2. Case Presentation

A 71-year-old Caucasian male was admitted to our hospital with severe acute kidney injury (AKI) and multiple overt bleedings. His medical history included hypertension, chronic obstructive pulmonary disease, obesity, heavy smoking, obstructive sleep apnoea, and hypercholesterolemia. In May 2017, the patient underwent pacemaker implant due to atrioventricular block; one month later, prolonged episodes of parossistic atrial fibrillation were recorded. Since the renal function was normal, serum creatinine (sCr) 0.97 mg/dL and estimated glomerular filtration rate (eGFR) 78 ml/min/1.73 m^2^, a direct oral anticoagulant (DOAC), i.e., dabigatran 150 mg twice a day, was started.

In March 2018, the patient reported persistent fatigue after a flu-like syndrome and occasional episodes of haematuria, haemoptysis, and epistaxis. The blood tests performed on 26^th^ of March showed anaemia with haemoglobin (Hb) of 9.4 g/dL and severe renal failure with sCr 5.12 mg/dL; therefore, the patient was urgently referred to emergency service. In the emergency room on 29^th^ of March, sCr was 6.4 mg/dL, activated partial thromboplastin time was 70 seconds, international normalized ratio was 2.3, and Hb was 8 g/dL. Idarucizumab, the antidote to dabigatran, was readily administered. Chest CT-scan showed bilateral localized ground glass lesions partly with pseudonodular pattern, while no significant abnormalities were seen at abdominal sonography. Rapidly progressive glomerulonephritis with a lung-kidney syndrome was suspected, and tests for antineutrophil cytoplasmic antibodies (ANCA) and antiglomerular membrane antibodies (anti-GBMAb) were urgently performed. Shortly after ELISA confirmation of antimyeloperoxidase antibodies positivity (anti-MPO titre of 61.4 RU/mL), the patient was transferred, on 30^th^ of March, to our Nephrology Unit with a hypothesis of new onset ANCA-associated vasculitis (AAV). Based on this hypothesis, an i.v. bolus of methylprednisolone 100 mg was empirically given on 5^th^ of April and the day after. Renal ultrasound showed normal-sized kidneys, increased cortical echogenicity, and slight reduction in corticomedullary differentiation. Given the chest images are not entirely suggestive for haemorrhagic alveolitis, the absence of systemic signs and symptoms, the negativity of anti-GBMAb and the low levels of inflammatory markers (protein reactive C was 9 mg/L, normal range < 5 mg/L), and the hypothesis of AAV were revised. The morphology of urine red blood cells (RBC) confirmed glomerular haematuria. A renal biopsy was performed on 6^th^ of April when sCr reached its peak of 7.9 mg/dL, and coagulopathy was safely reversed. Few hours later, the patient developed loin pain and a large self-limiting perirenal haematoma. Renal biopsy showed complex features: mesangial matrix expansion and hypercellularity at glomerulus; prominent acute tubular injury with several obstructive RBC casts; interstitium with blood extravasation and moderate inflammation; arteriosclerosis; and arteriolar hyalinosis. Immunofluorescence studies showed 4 + positivity for IgA (Figure 2). Therefore, AAV was excluded, and IgA nephropathy (Oxford score M1, E1, S1, T0, and C0) with possibly iatrogenic acute tubular-interstitial damage was diagnosed.

**Figure 1:**
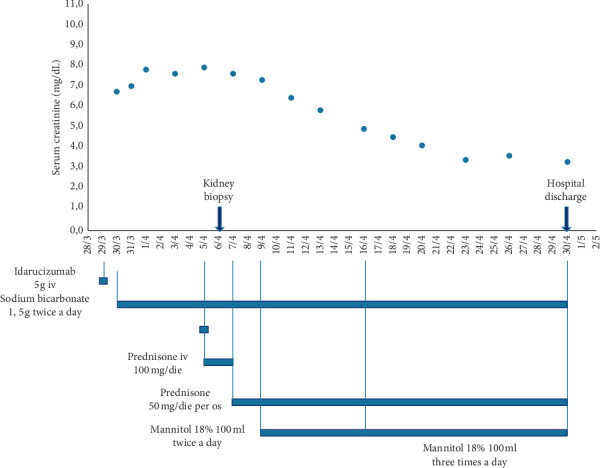
Kidney function trends and therapeutic strategies' timeline during hospitalization.

**Figure 2:**
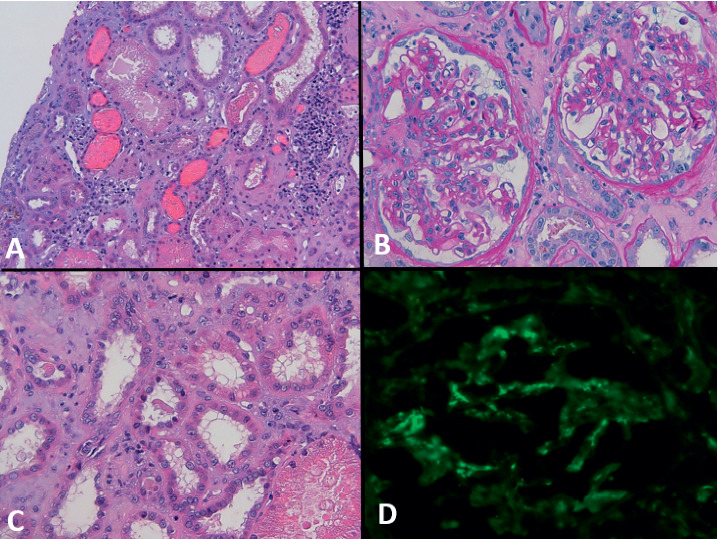
(A) Extensive tubular injury, partially haemolyzed and/or fragmented red blood cells within tubular lumen (H&E 10x). (B) In light microscopy, 10 glomeruli were identified; two glomeruli with mild expansion of mesangial matrix, minimal segmental endocapillary proliferation, flocculo-capsular adhesion with irregularities of Bowman's capsule, and activation of parietal epithelium; tubular injury (PAS 20x). (C) Tubular injury (H&E 20x). (D) IF stain for IgA with mesangial, vessels walls, and endotubular material staining pattern (20x).

To promote a “flushing” effect within tubules, mannitol was given from the 9^th^ of April onwards, with rapid and progressive decrease in sCr (Figure 1). Twenty-four hours urinary output was persistently above 1.5–1.8 litres, since the admission to hospital. Given the absence of plasma hyperosmolality and hyperosmolar hyponatremia and presuming mannitol effectiveness in maintaining osmotic diuresis, we decided to slightly increase mannitol dose from two to three times daily. Sodium bicarbonate was initially started for metabolic acidosis (arterial blood gas analysis showed a pH 7.34, pCO_2_ 38 mmHg, pO_2_ 85 mmHg, HCO_3_− 20 mmol/L, and BE −4 mmol/L), and subsequently maintained to force alkaline diuresis (urinary pH was increased from 5.0 and to 8.0 before discharge) and prevent occurrence of protein (haemoglobin) casts. Then, oral prednisone, 50 mg/day was associated and slowly tapered to 25 mg/day. The patients showed neither significant electrolyte disturbances nor symptoms/signs of uraemia and fluid overload. Balancing the potential risks of bleeding and infection related to central line placement and the benefits of intermittent haemodialysis in the lack of clinically relevant uraemia, renal replacement therapy (RTT) was not performed. The patient was discharged with sCr of 3.3 mg/dL, glomerular proteinuria 3.1 g/24 hours, and plenty of erythrocytes per high power field (HPF) at urine sediment.

Dabigatran was no longer restarted, and 4000 units/day of enoxaparin were introduced, until the patient underwent successful percutaneous closure of left atrial appendage in June. Renal function has progressively improved over time, and proteinuria and haematuria remarkably decreased. Prednisone was withdrawn in September 2018 when sCr was 1.2 mg/dL, proteinuria was 0.9 g/24h, and 1–5 RBC per HPF were detectable.

## 3. Discussion

After the introduction of direct oral anticoagulants OAC), few papers have reported AKI associated to their use, so the term “Anticoagulant-related nephropathy” (ARN) was coined to describe AKI caused by overanticoagulation [2].

Interestingly, it is suggested that overanticoagulation alone cannot provoke kidney damage. The main risk factors are glomerular overperfusion in the presence of reduced number of functioning nephrons and an underlying inﬂammatory glomerular disease [3]. In our patient, vascular and inflammatory factors together with advanced age could be the susceptible conditions for AKI, eventually triggered by overanticoagulation. We could not determine whether there was a preexisting kidney disease recognizable at the time of initiation of anticoagulation, as only sCr was available. The estimation of kidney function does not provide an exhaustive renal risk; urinalyses are useful to identify patients that deserve close attention during anticoagulation follow-up [4].

Generally, glomerular macrohematuria is considered a marker of glomerular basement membrane damage and inflammation [5] but, it can be also associated to AKI [6]; in fact, haematuria results in tubular injury in pigment-induced nephropathy (PIN), IgA nephropathy (IgAN), thin basement membrane nephropathy, and ARN [6, 7]. In vitro tubular cytotoxicity after RBC administration encouraged reevaluation of the predominant role of tubular obstruction in haematuria-associated renal damage. In fact, RBC is subjected to intratubular degradation and release heme-containing molecules and free iron [8], that have a direct oxidative cytotoxic effect on tubular cells. Overall, the pathogenesis of ARN is complex and multifactorial: significant erythrocytes filtration through an altered glomerular barrier is triggered by overanticoagulation and results in tubular obstruction and necrosis, inflammation, vasoconstriction-ischemia damage, proapoptosis, and oxidative stress [6]. This interplay between glomerulus and tubule in haematuria-associated renal damage recalls their interaction in many proteinuric glomerulonephritis. Consequently, the first question is whether the induction of inﬂammation and ﬁbrosis by haematuria promotes chronic kidney disease (CKD), likewise proteinuria. No prospective studies are available probably because ARN is still underestimated. However, given the growing evidence of progression of kidney disease in many haematuric conditions, the risk of CKD due to ARN remains a possibility [6]. A second issue is the importance of kidney biopsy in ARN. Considering the association between presumptive ARN with an increase in acute mortality rate [9], a rapid biopsy-proven diagnosis can be helpful [3]. Due to overanticoagulation and severe impairment of kidney function and coexisting comorbidities, renal biopsy is not always feasible. Although the coagulopathy was apparently reversed and the biopsy was performed by an expert physician, a clinically relevant bleeding occurred in our patient after procedure. Notably, this is not the only case of biopsy complicated by bleeding in the contest of dabigatran-induced nephropathy [10]. However, thanks to a biopsy-proven diagnosis, the management dramatically changed, RTT was avoided, and renal function rapidly improved.

To date, no guidelines for ARN treatment are available, and suggestions are gathered from case reports. Correction of coagulopathy (i.e., discontinuation of anticoagulant and administration of antidote) and general supportive care are the mainstays [11].The latter should include fluids administration and urine alkalinisation to prevent RBC precipitation in acidic environment. Early use of steroids to promote renal function recovery and minimize the risk for CKD is suggested by observational studies. Steroids were useful in our patient probably because of underlying IgAN; however, promoting an anti-inflammatory response could be beneficial also for haematuria-associated renal damage. Anti-inflammatory pathway induced by CD163 may decrease haemoglobin cytotoxicity and repair damaged tissues [12].

ARN and PIN share some pathogenetic features. Few evidence [7, 13] suggest that mannitol together with bicarbonate have no advantage over fluid resuscitation in managing AKI due to rhabdomyolysis, and its use remains controversial. However, randomized controlled trials are lacking, and its beneﬁcial effect cannot be excluded [14]. The rationale to use mannitol consists first in a washout effect, that flushes out intratubular necrotic debris [2]; second, in scavenger ability of hydroxyl and other free radicals [13], minimizing cells' injury through an antioxidant effect, and last, in vasodilatation and improvement of renal blood flow [15]. Although it is a long-standing drug, its benefits in haematuria-associated renal damage have not been established yet.

To the best of our knowledge, there are 8 published case reports describing biopsy-proven dabigatran-induced RBC cast nephropathy. Three patients needed RRT, with only two patients with eventually full recovery of renal function. Among patients conservatively treated, sCr returned to the baseline value in three patients only [10, 11, 16–21]. In the contest of overanticoagulation in a subject on NOAC, haematuria suggests that ARN should be taken into account in differential diagnosis; renal biopsy plays a crucial role in this field. None of these patients was treated with mannitol and bicarbonate; in our patient, the sharp and progressive decrease of sCr shortly after their initiation suggests that they could be an option.

Although warfarin has a stronger association with renal events compared to NOAC, several cases have highlighted safety issues for kidney related to NOAC use. In patients on anticoagulants presenting with AKI and haematuria, ARN should be always considered. Once coagulopathy is reversed, performing a kidney biopsy is important to identify any form of renal disease. Mannitol together with sodium bicarbonate and steroids was associated with rapid improvement in renal function. Although only a multicentric prospective study could establish the best strategy for ARN, the combination therapy described in the present case could be informative.
